# Facile Construction Engineering of Pr_6_O_11_@C with Efficient Photocatalytic Activity

**DOI:** 10.3390/molecules29153568

**Published:** 2024-07-29

**Authors:** Guoju Chang, Longzhong Ma, Yanhong Tu, Chenxin Mao, Paolo Aprea, Shiyou Hao

**Affiliations:** 1 Xingzhi College, College of Chemistry and Materials Science, Zhejiang Normal University, Jinhua 321004, China; 2 Department of Chemical, Materials and Production Engineering, University Federico II, P.le V. Tecchio 80, 80125 Naples, Italy

**Keywords:** Pr_6_O_11_@C, construction engineering, acid dye, ammonium hydroxide, photocatalytic activity

## Abstract

In this study, facile construction engineering of Pr_6_O_11_@C with efficient photocatalytic activity was established. Taking advantage of the flocculation of Pr^3+^ in the base medium, acid red 14 (AR14) was flocculated together with Pr(OH)_3_ precipitate, in which Pr(OH)_3_ and AR14 mixed highly uniformly. Calcinated at high temperature in N2, a novel Pr_6_O_11_@C was successfully synthesized. The resulting materials were characterized by XRD, SEM, FT-IR, Raman, and XPS techniques. The results show that the cubic Pr_6_O_11_@C with Fm3m space group, similar to that of Pr_6_O_11_, was obtained. From the results of the photodegradation of AR14, it is found that the photocatalytic efficiency of Pr_6_O_11_@C is higher than that of pure Pr_6_O_11_ due to the formation of abundant carbon bonds and oxygen vacancies. Compared with pure Pr_6_O_11_ and other carbon-based composites, the acid resistance of Pr_6_O_11_@C is greatly improved due to the highly uniform dispersion of Pr_6_O_11_ and C, which lays a solid foundation for the practical application of Pr_6_O_11_@C. Moreover, the role of NH3·H_2_O and NaOH used as precipitants for the photocatalytic efficiency of Pr_6_O_11_ was investigated in detail.

## 1. Introduction

Due to the complete degradation of organic pollutants, photocatalytic technology has received extensive attention [1,2,3]. In general, TiO_2_, ZnO, CuS et al. were often chosen as photocatalysts to degrade organic pollutants [4,5,6]. However, many difficulties, such as the large band gap for TiO_2_ and ZnO [7,8] and the photocorrosion problem for sulfide [9], should be overcome when using these photocatalysts. Therefore, there is an urgent need to develop other photocatalysts with lower band gaps and stable performance.

Recently, rare earth oxides such as CeO_2_ have attracted considerable attention due to their potential photocatalytic activity and their stability during photocatalysis [10,11,12]. Unfortunately, pure CeO_2_ has a large band gap of about 3.2 eV, which greatly decreases its absorption of visible light and hence photocatalytic efficiency. Pr_6_O_11_, an n-type semiconductor with a band gap of around 1.77–3.3 eV [13], is very stable among the praseodymium oxide family at ambient temperature and pressure [14]. Consequently, Pr_6_O_11_ was chosen as a photocatalyst to degrade organic pollutants [15,16,17]. In order to reduce the band gap, carbon materials were introduced into Pr_6_O_11_ to prepare the Pr_6_O_11_@C composite. For example, Shende et al. synthesized a new Pr_6_O_11_/g-C_3_N_4_ heterostructure material by a single-step solvent-free solid-state method and found that the notable increase in the photocatalytic activity of the Pr_6_O_11_/g-C_3_N_4_ heterostructure was ascribed to the reduced band gap and hence the improved visible light absorption [13]. However, the synthetic process of g-C_3_N_4_ is relatively complex, and the interaction between g-C_3_N_4_ and Pr_6_O_11_ is weak. Can g-C_3_N_4_ be replaced by other carbon materials during the synthesis of Pr_6_O_11_@C with efficient photocatalytic activity?

**Scheme 1:**
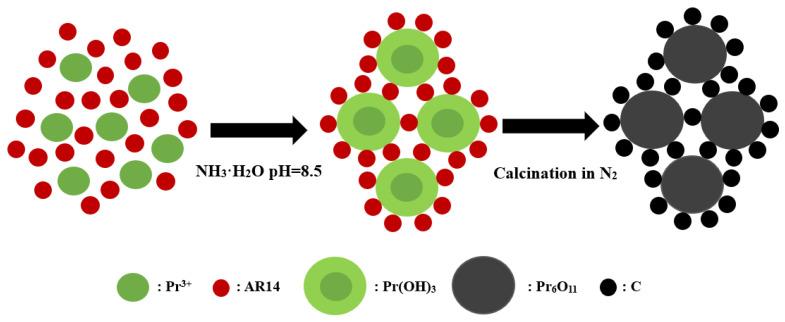
The scheme for the synthesis of Pr_6_O_11_@C.

Besides C_3_N_4_, activated carbon, carbon nanotube, grapheme, etc., are usually used as carbon sources to prepare carbon-based photocatalysts [18,19,20]. Nevertheless, the cost of carbon sources and the complex synthesis process of carbon-based catalysts hinder the practical application of these carbon materials. Moreover, these carbon-based Pr_6_O_11_ photocatalysts always react with H^+^ in an acid environment, which makes them unstable and limits their application in practice. Acid dyes containing rich carbon elements are always considered organic pollutants and removed by physical and/or chemical methods [21,22]. However, acid dyes are rarely used as carbon sources to synthesize carbon-based photocatalysts. Recently, it has been interesting to find that Pr^3+^ can flocculate acid red 14 (AR14) in the basic medium, which makes us consider the use of acid dyes such as AR14 as carbon sources for the preparation of Pr_6_O_11_@C. In the flocculation process, Pr^3+^ was precipitated into Pr(OH)_3_ by mixing with AR14 uniformly to form Pr(OH)_3_@AR14. Calcinated at high temperatures, Pr(OH)_3_ and AR14 can be changed into Pr_6_O_11_ and carbon, respectively. The synthetic process of Pr_6_O_11_@C based on this design may be illustrated in Scheme 1. To the best of our knowledge, there are no reports about the synthesis of Pr_6_O_11_@C via this facile construction engineering.

Guided by the above idea, Pr_6_O_11_@C was synthesized using acid dye as a carbon source and Pr^3+^ as a flocculatant. The photocatalytic performance of Pr_6_O_11_@C was measured by the degradation of AR14, and the results show that the photocatalytic efficiency of Pr_6_O_11_@C is higher than that of pure Pr_6_O_11_ due to the uniform mixing of carbon with Pr_6_O_11_ and the efficient carbon bond formation rate. Accordingly, the acid resistance of Pr_6_O_11_ was greatly improved. Furthermore, the effect of different precipitants (NH_3_·H_2_O and NaOH) on the photocatalytic efficiency was also investigated.

## 2. Experimental

The experimental part is presented in the Supporting Materials.

## 3. Results and Discussion

**Figure 1:**
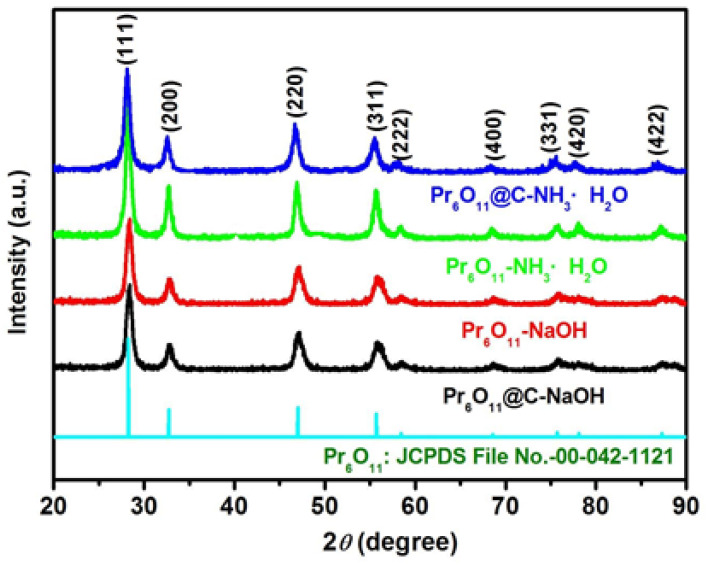
XRD patterns of Pr_6_O_11_ and Pr_6_O_11_@C prepared with different precipitant.

Figure 1 shows the powder X-ray diffraction (XRD) patterns of Pr_6_O_11_ and Pr_6_O_11_@C prepared with different precipitants. The diffraction pattern of the synthesized samples can be ascribed to the cubic Pr_6_O_11_ with the *Fm3m* space group (JCPDS file No. 00-042-1121) [16]. It is clear from Figure 1 that all samples exhibit obvious peaks corresponding to the (111), (200), (220), (311), (222), (400), (331), (420), and (422) planes, indicating that both the precipitant and the introduction of carbon have no effect on the crystal structure of the prepared samples.

**Figure 2:**
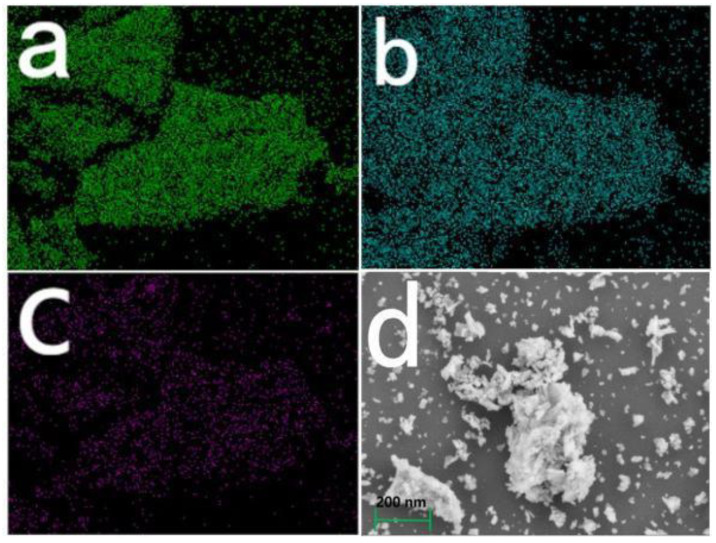
Elemental mapping of (**a**) O, (**b**) Pr, (**c**) C and SEM image (**d**) of Pr_6_O_11_@C prepared using 100 mL of AR14 with a concentration of 0.075 mM as carbon source.

The elemental mapping of O, Pr, and C from EDS is presented in Figure 2, and the results show that C was actually introduced into the resulting sample and that all the composited elements were evenly distributed in the sample. Similar phenomena can be found in other samples with different content of carbon, illustrating that AR14 can be changed into carbon in our experimental process.

**Figure 3:**
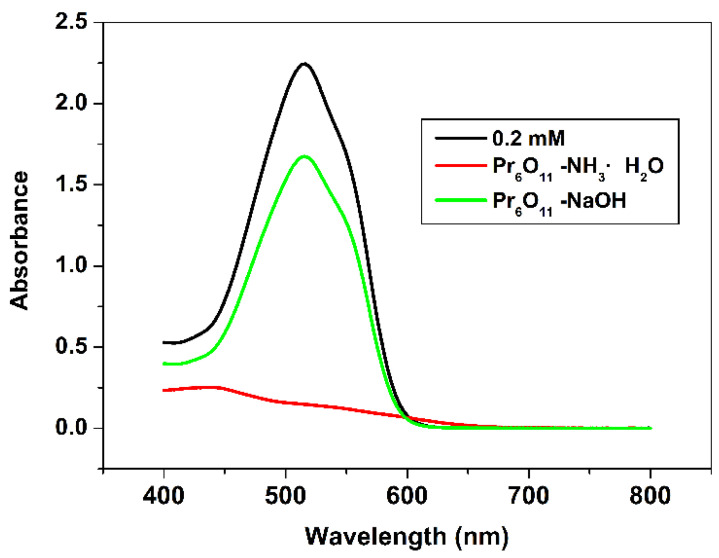
UV-vis spectra of 0.2 mM AR14 after photodegradation over Pr_6_O_11_-NH_3_·H_2_O and Pr_6_O_11_-NaOH.

The photocatalytic efficiency of Pr_6_O_11_-NH_3_·H_2_O and Pr_6_O_11_-NaOH was evaluated by the degradation of AR14, which is presented in Figure 3. From Figure 3, it is easy to find that the photocatalytic efficiency of Pr_6_O_11_-NH_3_·H_2_O is much higher than that of Pr_6_O_11_-NaOH because the characteristic absorption peak intensity of AR14 at 516 nm is very low after degradation over Pr_6_O_11_-NH_3_·H_2_O. From the results of XRD, the structure of Pr_6_O_11_-NH_3_·H_2_O and Pr_6_O_11_-NaOH is similar. Why are the photocatalytic results so different?

**Figure 4:**
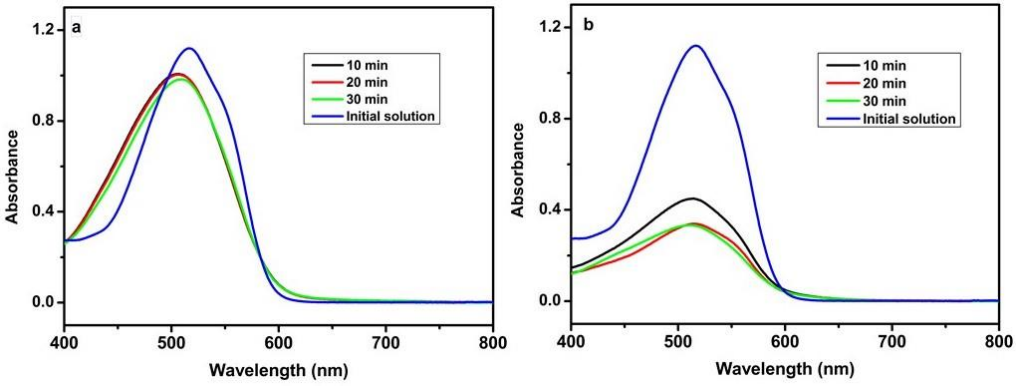
UV-vis spectra of 0.1 mM AR14 after adsorption over Pr_6_O_11_-NaOH (**a**) and Pr_6_O_11_-NH_3_·H_2_O (**b**).

It is well known that the adsorption capability of organic pollutants over the catalyst plays an important role in photocatalytic efficiency [23]. In order to investigate the intrinsic reason for photocatalytic difference, the adsorption of 0.1 mmol/L (mM) AR14 on Pr_6_O_11_-NH_3_·H_2_O and Pr_6_O_11_-NaOH was carried out, and the results are shown in Figure 4. Compared with Figure 4a,b, it is obvious that the adsorption efficiency of Pr_6_O_11_-NH_3_·H_2_O is higher than that of Pr_6_O_11_-NaOH and that 30 min is enough for the adsorption equilibrium of AR14 over Pr_6_O_11_-NH_3_·H_2_O and Pr_6_O_11_-NaOH. From our previous report, it can be seen that the number of amino groups and hydroxyl groups in the sample prepared using NH_3_·H_2_O as the precipitant is higher than that of the sample synthesized using NaOH as the precipitant, which results in a higher adsorption efficiency [24].

**Figure 5:**
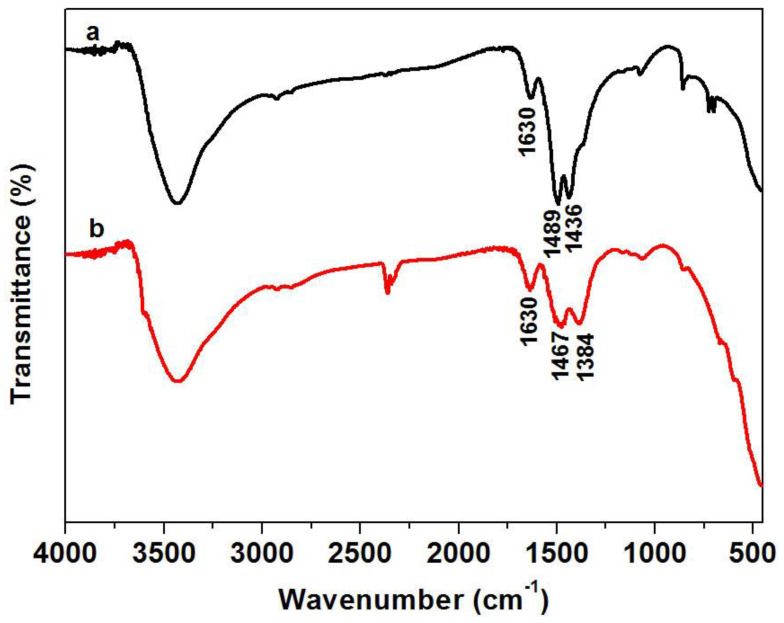
FTIR spectra of Pr_6_O_11_-NaOH (a) and Pr_6_O_11_-NH_3_·H_2_O (b).

In order to investigate if there are amino groups and hydroxyl groups in Pr_6_O_11_-NaOH and Pr_6_O_11_-NH_3_·H_2_O, FT-IR was used, and the results are presented in Figure 5. It can be found from Figure 5 that amino groups and hydroxyl groups actually reside in Pr_6_O_11_-NH_3_·H_2_O because of the peaks at 1630 cm^−1^ ascribed to the O-H bending vibration [25] and 1467 cm^−1^ attributed to the N-H bending vibration mode [26]. The peak at 1630 cm^−1^ is also detected in Pr_6_O_11_-NaOH, but the intensity is lower than that of Pr_6_O_11_-NH_3_·H_2_O, implying that the hydroxyl content of Pr_6_O_11_-NH_3_·H_2_O is higher than that of Pr_6_O_11_-NaOH. According to the relevant literature [27,28,29], the peaks at 1489, 1436, and 1384 cm^−1^ are due to the stretching vibration of the redundant ${\mathrm{NO}}_{3}^{-}$ on the surface of the prepared samples. From Figure 5, it can be inferred that the number of ${\mathrm{NO}}_{3}^{-}$ in Pr_6_O_11_-NaOH is higher than that of Pr_6_O_11_-NH_3_·H_2_O because two peaks representing ${\mathrm{NO}}_{3}^{-}$ are detected on a curve, while only one characteristic ${\mathrm{NO}}_{3}^{-}$ peak is found on curve b. Consequently, the reasons for the higher adsorption efficiency of AR14 over Pr_6_O_11_-NH_3_·H_2_O can be presented as follows. On one hand, there are lots of hydroxyl and amino groups on the surface of Pr_6_O_11_-NH_3_·H_2_O, resulting in strong interaction between Pr_6_O_11_-NH_3_·H_2_O and AR14 due to the electrostatic attraction of sulfo groups (-${\mathrm{SO}}_{3}^{-}$) and -${\mathrm{OH}}_{2}^{+}$ and ${\mathrm{NH}}_{4}^{+}$ in the acid system [24]. On the other hand, less content of ${\mathrm{NO}}_{3}^{-}$ in Pr_6_O_11_-NH_3_·H_2_O can decrease the repulsive force between ${\mathrm{NO}}_{3}^{-}$ and -${\mathrm{SO}}_{3}^{-}$. Moreover, the existence of ${\mathrm{NO}}_{3}^{-}$ on the surface of the photocatalyst can decrease the content of radicals, such as hydroxyl radicals, due to the capture of ${\mathrm{NO}}_{3}^{-}$ for radicals [30,31], which can also explain the lower photocatalytic efficiency of Pr_6_O_11_-NaOH.

**Figure 6:**
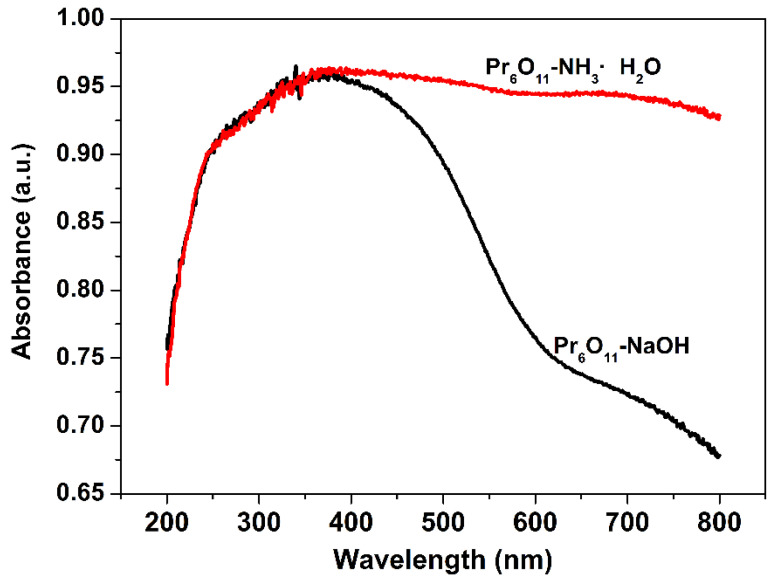
Diffuse reflectance UV-vis spectra of Pr_6_O_11_-NaOH and Pr_6_O_11_-NH_3_·H_2_O.

The light, especially for visible light, absorption efficiency of photocatalysts is another important factor affecting photocatalytic efficiency. From the diffuse reflectance UV-vis spectra of Pr_6_O_11_-NaOH and Pr_6_O_11_-NH_3_·H_2_O (Figure 6), it can be seen that the absorption efficiency of visible light over Pr_6_O_11_-NH_3_·H_2_O is much higher than that of Pr_6_O_11_-NaOH, implying that photogenerated electrons and holes can be efficiently produced and, hence, photocatalytic efficiency will be improved when Pr_6_O_11_-NH_3_·H_2_O is used as a photocatalyst.

**Figure 7:**
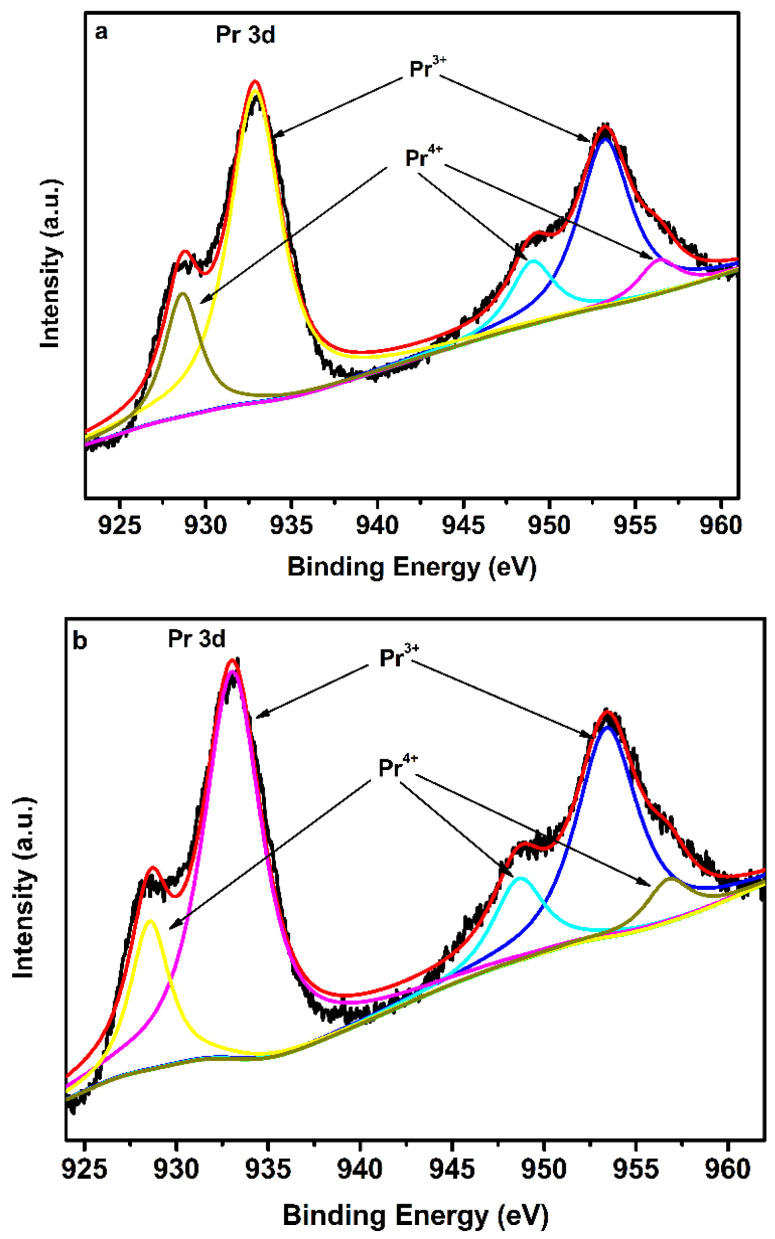
XPS spectra of Pr 3d for Pr_6_O_11_-NaOH (**a**) and Pr_6_O_11_-NH_3_·H_2_O (**b**).

As discussed above, Pr^3+^ and Pr^4+^ are always detected in Pr_6_O_11_, and the ratio of Pr^4+^ to Pr^3+^ can affect the photocatalytic efficiency of Pr_6_O_11_. The content of Pr^4+^ and Pr^3+^ in Pr_6_O_11_-NaOH and Pr_6_O_11_-NH_3_·H_2_O can be defined by the XPS spectra of Pr 3d, which is shown in Figure 7. The Pr 3d spectra in Figure 7a,b can be deconvoluted into five peaks centered at 933, 953, 948, 928, and 956 eV, respectively. The strong peaks at 933 and 953 eV correspond to Pr^3+^, and the other peaks are ascribed to Pr^4+^ [32,33]. According to the peak area of Pr^4+^ and Pr^3+^ in Figure 7, it can be concluded that the ratio of Pr^4+^ to Pr^3+^ in Pr_6_O_11_-NaOH (0.27) is higher than that of Pr_6_O_11_-NH_3_·H_2_O (0.25), implying less content of Pr^4+^ in Pr_6_O_11_-NH_3_·H_2_O. Therefore, we can conclude that more Pr^4+^ ions on the surface of Pr_6_O_11_-NH_3_·H_2_O were reduced to Pr^3+^, and thus more oxygen vacancies were formed. According to Gregson et al., trivalent lanthanide ions make it easier to form lanthanide carbon bonds than that of tetravalent ones [34]. Generally, the easy formation of carbon bonds in the photocatalyst is helpful for photocatalytic efficiency [35]. Moreover, oxygen vacancies can improve the separation of photogenerated electrons and holes, resulting in more efficient photocatalytic efficiency [36], which can be proved by the results of photocurrent over Pr_6_O_11_-NaOH and Pr_6_O_11_-NH_3_·H_2_O (Figure 11b). From the analysis of XPS spectra of Pr 3d, the results in Figure 3 can be further understood.

**Figure 8:**
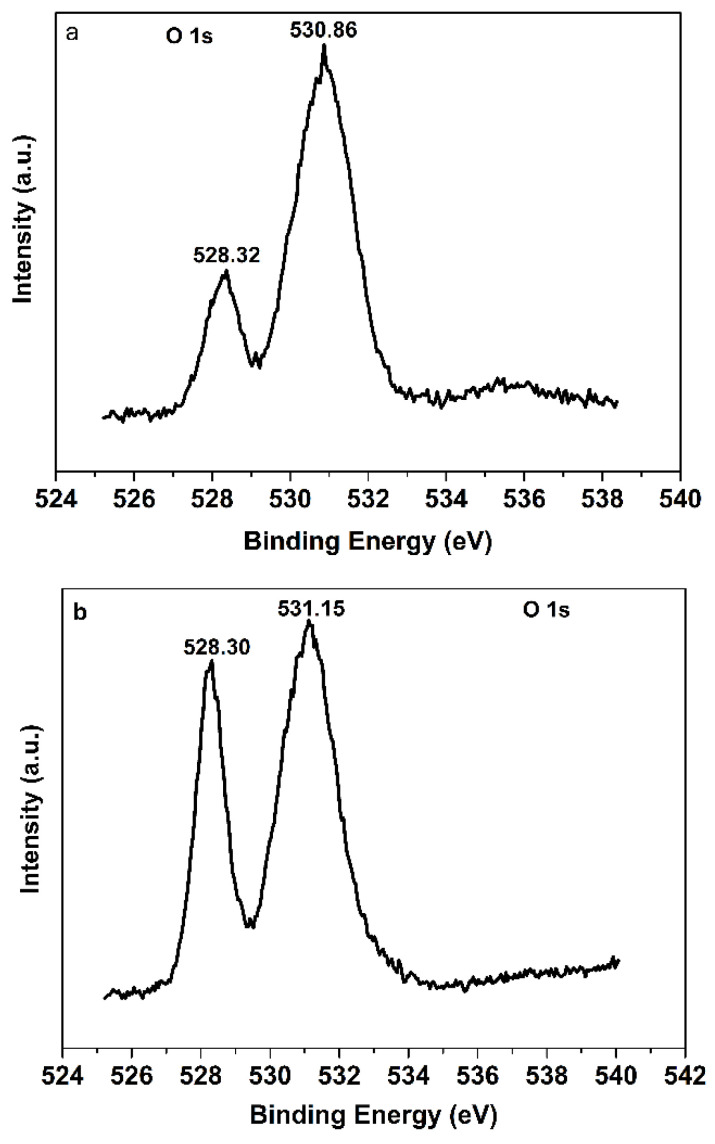
XPS spectra of O 1s for Pr_6_O_11_-NaOH (**a**) and Pr_6_O_11_-NH_3_·H_2_O (**b**).

Besides the structural information given by XPS spectra of Pr 3d, XPS spectra of O 1s can also provide useful messages for the structure of Pr_6_O_11_. From XPS spectra of O 1s for Pr_6_O_11_-NaOH and Pr_6_O_11_-NH_3_·H_2_O (Figure 8), it can be inferred that the strength of the Pr-O bond in Pr_6_O_11_-NH_3_·H_2_O is higher than that in Pr_6_O_11_-NaOH because the peak area at about 528.30 and the characteristic binding energy of Pr-O [33] in Pr_6_O_11_-NH_3_·H_2_O are higher than those of Pr_6_O_11_-NaOH. A stronger Pr-O bond can make Pr_6_O_11_-NH_3_·H_2_O more stable, which is beneficial for its practical application. Moreover, more Pr-O bonds may be helpful for the transition of photogenerated electrons to the surface of the catalyst to combine the adsorbed oxygen and form a superoxide radical to degrade AR14. The peak at about 531 eV in Figure 8 can be attributed to oxygen species in the defects [37] and adsorbed oxygen [38]. Due to the high intensity, the peak at about 531 eV is mainly assigned to the adsorbed oxygen. It is clear from Figure 8 that the ratio of adsorbed oxygen to binding oxygen in Pr_6_O_11_-NaOH is higher than that in Pr_6_O_11_-NH_3_·H_2_O. It can be concluded from the literature [39] that the high content of adsorbed oxygen can result in poor activity of the catalyst, which can further prove the lower photocatalytic efficiency of Pr_6_O_11_-NaOH.

**Figure 9:**
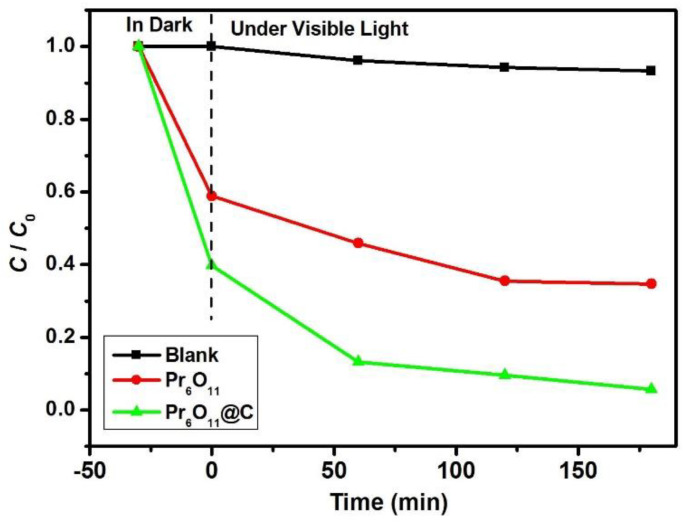
The photodegradation of 0.3 mM AR14 in the presence of Pr_6_O_11_ and Pr_6_O_11_@C under visible light irradiation.

**Figure 10:**
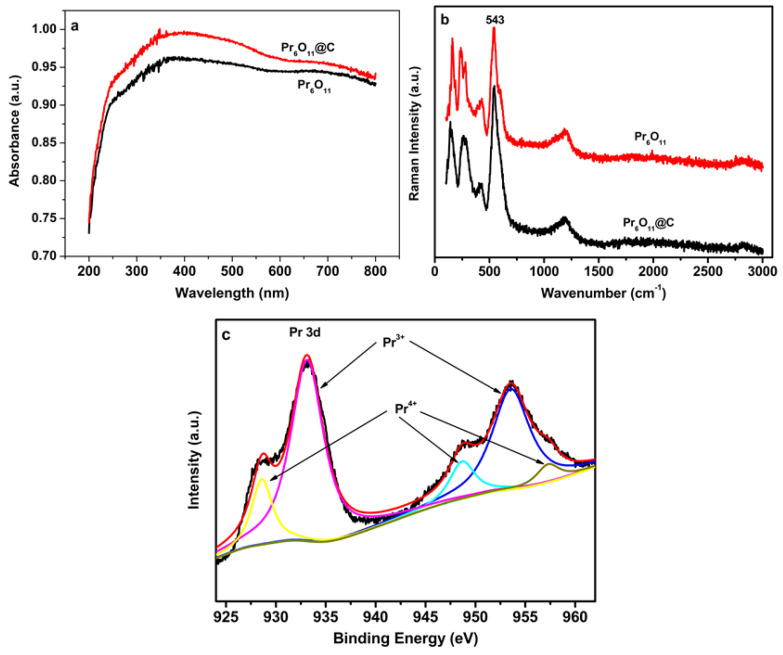
Diffuse reflectance UV-vis spectra (**a**), Raman spectra of Pr_6_O_11_ and Pr_6_O_11_@C (**b**), and XPS spectra of Pr 3d for Pr_6_O_11_@C (**c**).

From the above discussion, it can be concluded that NH_3_·H_2_O is suitable for the preparation of Pr_6_O_11_ with enhanced photocatalytic efficiency. Therefore, NH_3_·H_2_O is designated as a precipitant to synthesize Pr_6_O_11_ and Pr_6_O_11_@C in the following context. The photodegradation of 0.3 mM AR14 over Pr_6_O_11_ and Pr_6_O_11_@C prepared via the route of Scheme 1 is illustrated in Figure 9. It is obvious that the introduction of carbon into Pr_6_O_11_ can improve the photocatalytic efficiency of Pr_6_O_11_ under the same experimental conditions. From the dark reaction, it is easy to find that the adsorption capacity of Pr_6_O_11_@C is higher than that of the pure composite, which is good for the enhancement of photocatalytic efficiency. Furthermore, it can be found from Figure 10a that, compared with Pr_6_O_11_, the UV-vis absorption of Pr_6_O_11_@C is more efficient, which is helpful for the improvement in photocatalytic activity. From Figure 10b, we can find that the intensity of peak at 543 nm, associated with Pr^3+^ and oxygen vacancy (positively charged) [40], of Pr_6_O_11_@C is higher than that of Pr_6_O_11_, implying more efficient photocatalysis of Pr_6_O_11_@C because the photogenerated electrons can be easily captured by the positively charged oxygen vacancies [34,36]. The existence of Pr^3+^ and the oxygen vacancy in Pr_6_O_11_@C is further demonstrated by the XPS spectra of Pr 3d for Pr_6_O_11_@C (Figure 10c) because the ratio of Pr^4+^ to Pr^3+^ is 0.22, which is lower than that of Pr_6_O_11_ (0.25). A possible reason for this is that the carbon produced by the pyrolysis of AR14 is active [35] and can reduce Pr^4+^ to Pr^3+^, resulting in the formation of oxygen vacancies via the replacement of Pr^4+^ by Pr^3+^.

**Figure 11:**
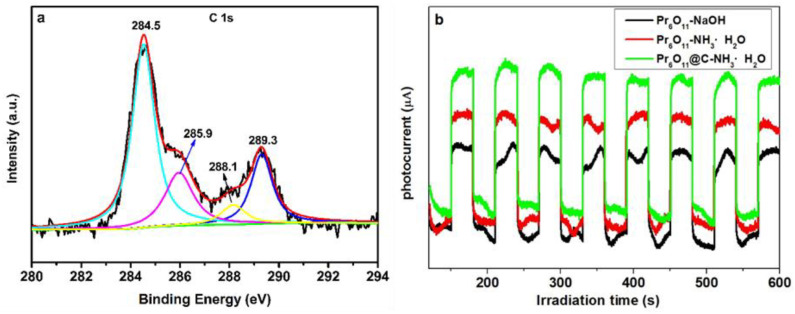
XPS spectra of C 1s for Pr_6_O_11_@C (**a**), and photocurrent–time profiles of Pr_6_O_11_-NaOH, Pr_6_O_11_-NH_3_·H_2_O, and Pr_6_O_11_@C-NH_3_·H_2_O (**b**).

From our previous report, the formation of carbon bonds between carbon and photocatalyst can greatly improve photocatalytic efficiency [35]. The carbon bonding in Pr_6_O_11_@C can be investigated by XPS spectra of C 1s, which is presented in Figure 11a. The C1s spectra in Figure 11a can be deconvoluted into four peaks centered at 284.5, 285.9, 288.1, and 289.3 eV corresponding to C=C, C-OH, O=C and O-C=O bonds, respectively [41]. Consequently, it can be concluded that numerous carbon bonds were formed in Pr_6_O_11_@C, which can enhance the separation of photogenerated electrons and holes. In order to test this inference, a photocurrent experiment was carried out, and the results are shown in Figure 11b. It is obvious from Figure 11b that the photocurrent on Pr_6_O_11_@C is higher than that on Pr_6_O_11_, indicating more efficient photoelectron–hole separation efficiency for Pr_6_O_11_@C. It can be concluded from Figure S1 that AR14 was actually degraded over Pr_6_O_11_@C. From the results of Figure S2, it is obvious that 0.075 mM AR14 is the best concentration to synthesize Pr_6_O_11_@C with efficient photocatalytic efficiency. Moreover, Figure S3 proves that $O_{2}^{•-}$ and OH• are the main oxidative species responsible for the photodegradation of AR14.

**Figure 12:**
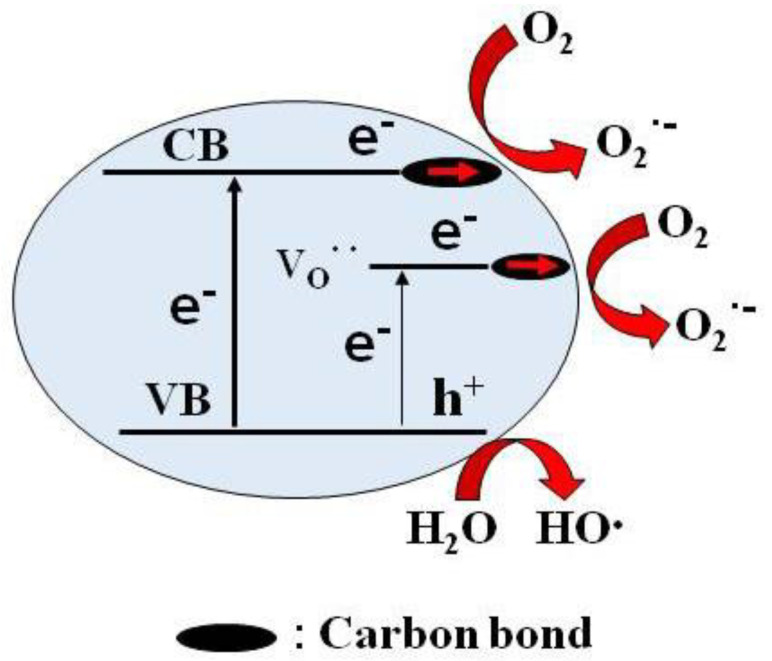
Proposed photocatalytic mechanism over Pr_6_O_11_@C-NH_3_·H_2_O under the irradiation of visible light ($V_{O}^{••}$: oxygen vacancy, h^+^: hole, VB: valence band, CB: conduction band).

According to the above analysis, it can be concluded that lots of oxygen vacancies and carbon bonds were formed in the Pr_6_O_11_@C, and O_2_ can be adsorbed on the surface of Pr_6_O_11_@C. Therefore, under visible light irradiation, the electrons can be excited from VB to CB, intermediate energy levels resulting from oxygen vacancies, and transfer carbon bond and reach the surface of Pr_6_O_11_@C to combine with the adsorbed oxygen to form $O_{2}^{•-}$. On the other hand, the holes formed after the departure of excited electrons can interact with water to form OH• due to their strong oxidation. $O_{2}^{•-}$ and OH• degrade AR14 together. Therefore, the possible photocatalytic mechanism of AR14 over Pr_6_O_11_@C can be illustrated by the schematic diagram of the energy band of Pr_6_O_11_@C, as shown in Figure 12.

**Figure 13:**
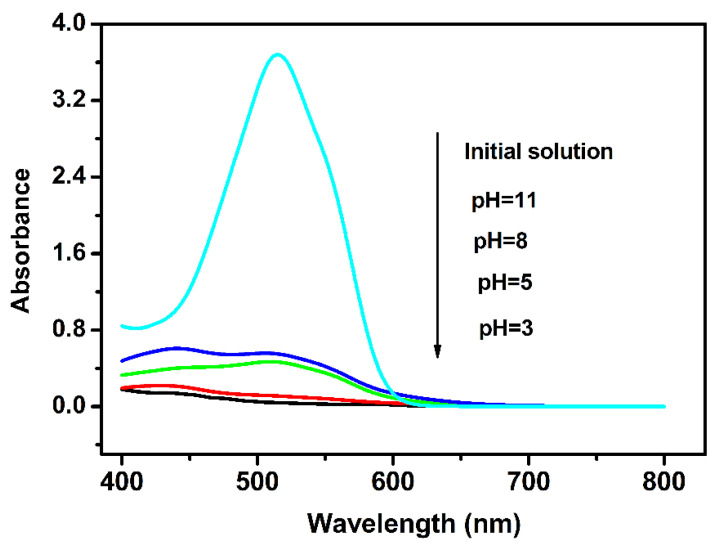
Effect of pH on the degradation of 0.3 mM AR14 over Pr_6_O_11_@C for 180 min.

From the viewpoint of practical application, a stable photocatalyst in a medium with different pH values is welcome. The effect of pH on the degradation of 0.3 mM AR14 over Pr_6_O_11_@C is shown in Figure 13. It is clear from Figure 13 that Pr_6_O_11_@C is stable in both acidic and alkaline media and that the photocatalytic efficiency of Pr_6_O_11_@C in an acid medium is higher than that of an alkaline medium. The reason for this may be that the adsorption ability of acid dyes over photocatalysts is increased in an acid medium [40]. It can be seen from Figure 13 that AR14 is almost degraded in the system with a pH of 5, and the degradation rate of AR14 is not obviously increased when the pH value is decreased to 3. From the experimental results, we found that pure Pr_6_O_11_ can be dissolved in the solution with a pH value of 4 because of an acid-base reaction. However, the prepared Pr_6_O_11_@C in this study is stable in the solution with a pH value of 3. In order to investigate the stability of other carbon-based Pr_6_O_11_ in acid solution, we synthesized Pr_6_O_11_@C using C_3_N_4_, carbon nanotube, and grapheme as carbon source. The preparation process is similar to Pr_6_O_11_@C prepared using AR14 as a carbon source. The AR 14 solution was replaced by a 100 mL solution containing 0.01 g C_3_N_4_ or carbon nanotube or grapheme. The results show that these carbon-based Pr_6_O_11_ materials are not stable in the solution with a pH value of 4, indicating that using AR14 as a carbon source is helpful for the stability of Pr_6_O_11_@C in the acid medium and that Pr_6_O_11_@C synthesized via Scheme 1 is a potential photocatalyst. A possible reason for the acid resistance of Pr_6_O_11_@C synthesized in this study is that carbon and Pr_6_O_11_ are mixed highly uniformly with each other, which can be seen from Scheme 1.

**Figure 14:**
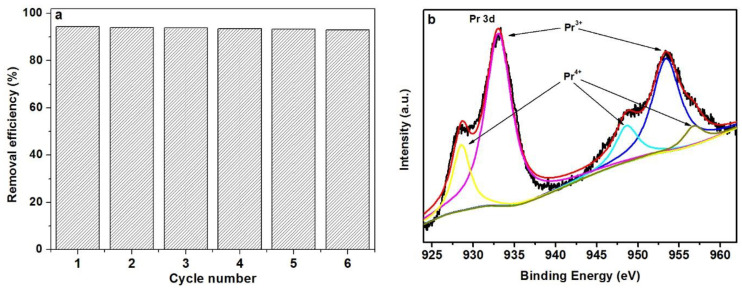
Reuse performance (**a**) and XPS spectra of Pr 3d for Pr_6_O_11_@C after six photocatalytic cycles (**b**).

From the results of Figure 14a, it can be seen that Pr_6_O_11_@C demonstrates good photocatalytic stability because the sixth photocatalytic efficiency is similar to the first one. Moreover, the Pr^3+^ and Pr^4+^ are detected in Pr_6_O_11_@C after six cycles (Figure 14b), which is the same as that of the initial sample.

## 4. Conclusions

Pr_6_O_11_@C with efficient photocatalytic efficiency and acid resistance was prepared via facile construction engineering. As a precipitant, NH_3_·H_2_O is more suitable for the preparation of Pr_6_O_11_ with enhanced photocatalytic activity than that of NaOH because more amino groups and hydroxyl groups were detected in the sample synthesized using NH_3_·H_2_O as a precipitant. Moreover, the absorption intensity of visible light and the ratio of Pr^3+^ in Pr_6_O_11_-NH_3_·H_2_O are higher than those of Pr_6_O_11_-NaOH. Due to the formation of carbon bonds and oxygen vacancies, Pr_6_O_11_@C prepared using NH_3_·H_2_O as a precipitant has more efficient photocatalytic activity compared with that of the pure composite. The optimum one is 0.075 mM AR14 for synthesizing Pr_6_O_11_@C. $O_{2}^{•-}$ and OH• are the main oxidative species responsible for the photodegradation of AR14. The use of AR14 as a carbon source is helpful for the stability of Pr_6_O_11_@C in the acid medium. Pr_6_O_11_@C had good photocatalytic stability, indicating that it has the potential application for the removal of organic pollutants.

## Data Availability

Data are contained within the article and Supplementary Materials.

## Supplementary Materials

The following supporting information can be downloaded at: https://www.mdpi.com/article/10.3390/molecules29153568/s1. Refs. [35,42] are cited in Supplementary Materials.
