# Supplementary material for: Facile Construction Engineering of Pr6O11@C with Efficient Photocatalytic Activity
Source: Molecules. 2024 Jul 29;29(15):3568. doi: 10.3390/molecules29153568 (PMC11314047; doi:10.3390/molecules29153568)
Supplement: Supplementary file 1 [file molecules-29-03568-s001.zip › molecules-2987882-supplementary.pdf]

A Facile Construction Engineering of Pr_6O_{11}@C with Efficient Photocatalytic Activity

Guoju Chang [a #], Longzhong Ma [a #], Yanhong Tu [a], Chenxin Mao[a], Paolo Aprea [b], Shiyou Hao [a,*]

[a]Xingzhi College, College of Chemistry and Life Sciences, Zhejiang Normal University, 321004 Jinhua，P. R. of China

[b]Department of Chemical, Materials and Production Engineering, University Federico II, P.le V. Tecchio 80, 80125, Naples, Italy

[#] Guoju Chang and Longzhong Ma, equally contributed to this paper, are the co-first authors.

*Corresponding author:

E-mail: sky54@zjnu.cn (Shiyou Hao)

1. Experimental details

1.1 *Chemicals and synthesis*

Praseodymium (III) nitrate hexahydrate ($Pr(NO_3)_3·6H_2O$), KI, 1, 4-benzoquinone (BQ), terephthalic acid (TPA), ammonium hydroxide ($NH_3·H_2O$, 27%), sodium hydroxide (NaOH), absolute ethanol, and 37% fuming hydrochloric acid were purchased from Sinopharm Chemical Reagent Co. All the chemical reagents were used without further purification. Deionized water with a resistivity larger than 18.2 MΩ was obtained from Millipore Milli-Q® ultrapure water purification systems. 0.2 M of HCl solution was prepared from the 37% fuming hydrochloric acid. Different concentration of AR 14 and 0.2 M of NaOH solutions were obtained by dissolved AR14 (Sigma-Aldrich) and NaOH in deionized water at room temperature, respectively. The pH values of all the solutions containing AR14 used for the adsorption experiments were adjusted to the desired ones using 0.2 M HCl or 0.2 M NaOH solution.

Typically, the synthesis of Pr_6O_{11}@C can be described as follows: 1 g of $Pr(NO_3)_3·6H_2O$ was dissolved into 100 mL of AR14 solution with different concentration at room temperature under stirring. No special instruction, the concentration of AR14 used for the preparation of Pr_6O_{11}@C is 0.075 mM. After the solution became clear, a certain amount of ammonium hydroxide ($NH_3·H_2O$, 27%) or 0.2 M NaOH solution was added into the solution to adjust solution pH to 8.5 at room temperature. At this point, a red floccule was formed in the solution. Afterwards, the precipitate was aged for 3 h. The red solid was then recovered by filtration, washed with water and ethanol for three times, and dried at 60 ℃ for 2 h. Finally, the red solid was calcined in N_2 at 600 °C for 3 h with a heating ramp of 5 °C min^{-1}

to synthesize Pr_6O_{11}@C composite. Pr_6O_{11} was synthesized with the same method as that of Pr_6O_{11}@C except that 100 mL of AR14 solution with different concentration was displaced by 100 mL of deionized water. According to the precipitant used in the synthetic product, the obtained composites are hereafter denoted as Pr_6O_{11}-$NH_3 \cdot H_2O$, Pr_6O_{11}-NaOH, and Pr_6O_{11}@C-$NH_3 \cdot H_2O$, Pr_6O_{11}@C-NaOH.

1.2 *Characterization*

The X-ray diffraction (XRD) patterns were collected on a Philips PW3040/60 powder diffractometer using CuKα radiation (λ = 0.154 nm). The FT-IR spectra were recorded by a Nicole Nexus 670 spectrometer with a resolution of 4 cm^{-1} using the KBr pellet method. Elemental mapping was taken on the FE-SEM using a Hitachi S-4800 instrument operating at 50 kV. The diffuse reflectance spectra of the samples over a range of 200-800 nm were recorded by a Nicolet Evolution 500 Scan UV-vis system. Raman scattering analysis was performed on a Renishaw RM1000 Raman spectrometer with a 514 nm excitation laser light. X-ray photoelectron spectroscopy (XPS) measurement was carried out on a RBO upgraded PHI-5000 C ESCA system (Perkin Elmer) using monochromated Al Kα X-rays (E=1486.6 eV) as a radiation at 250 W. All binding energies were calibrated using carbon (C_{1S} = 284.6 eV) as a reference. The photoluminescence (PL) spectra of the samples were obtained at room temperature by a spectrofluorometer (NanoLOG-TCSPC, Horiba Jobin Yvon, USA) with an excitation wavelength of 250 nm. The adsorption isotherms of N_2 were measuredwith a Micromeritics ASAP 2020 apparatus at -196 ^{o}C and the specific surface areas of the investigated samples were calculated using the multiple-point Brunauer-Emmett-Teller (BET) method in the relative pressure range p/p_0 = 0.05-0.3. The

electrochemical tests were carried out with a CH Instruments CHI660D electrochemical station in a standard three electrode configuration.

1.3 *Photocatalytic tests*

The photocatalytic activities of Pr_6O_{11}@C and Pr_6O_{11} samples were evaluated by the degradation of AR14. Typically, 20 mL 0.2 mM or 0.3 mM of AR14 solution and 20 mg of photocatalyst powder were placed in a beaker under vigorous agitation at pH value of initial AR14 solution (about 6.0). Afterwards, the suspensions were stirred in the dark for 30 min to reach the adsorption-desorption equilibrium. Then the mixed solution was continually stirred under the irradiation of the visible light (410-750 nm). At given time intervals, a small amount of suspension was withdrawn and centrifuged to remove the photocatalyst. The residual AR14 levels in the filtrates were then analyzed by recording the variations of the absorbance at 516 nm with a UV-vis spectrophotometer (Evolution 500LC). The degradation efficiency of AR14 was evaluated as C/C_o, where C_0 is the initial concentration of AR14 (mmol/L) and C is the concentration of AR14 (mmol/L) in the filtrates.

The pH value of photocatalytic system was adjusted by 0.2 M HCl (aq) or 0.2 M NaOH (aq). The photocatalysts used for the photocatalytic stability were recovered from the sampless generated after photocatalysis via filtration and drying. The steps of the repeated photocatalytic experiments are same as those of non repeated experiments.

2. Results and discussion

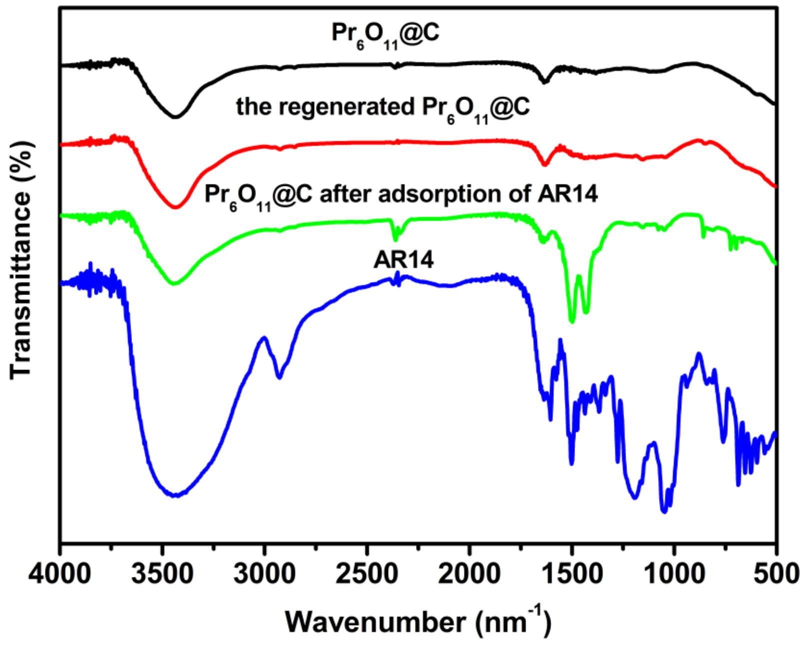

Fig. S1 FT-IR spectra of AR14, Pr_6O_{11}@C after adsorption of AR14, Pr_6O_{11}@C and the regenerated Pr_6O_{11}@C.

In order to make sure that the removal of AR14 from solution is attributed to adsorption or photocatalysis, FT-IR spectra of AR14, Pr_6O_{11}@C after adsorption of AR14, Pr_6O_{11}@C and the regenerated Pr_6O_{11}@C was performed and the results are presented in Fig. S1. It can be seen that AR14 was mainly adsorbed on the catalyst in the dark reaction because the characteristic absorption bands of AR14 were found in the FT-IR spectra of Pr_6O_{11}@C and that AR14 was really degraded under the irradiation of visible light due to no detection of AR14 over the regenerated Pr_6O_{11}@C.

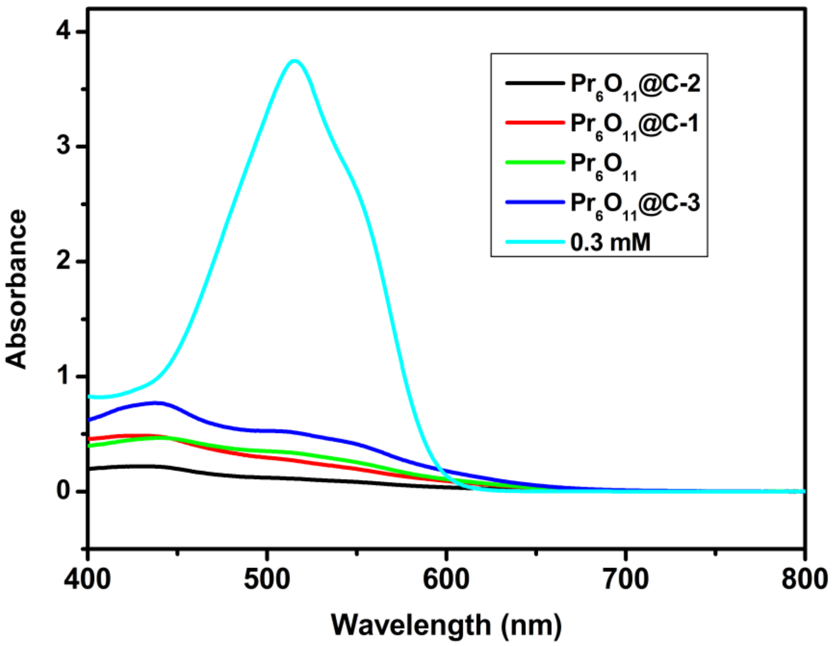

Fig. S2 Effect of carbon content on the degradation of 0.3 mM AR14 over Pr_6O_{11}@C for 180 min.

From the previous report (Wang er al. 2020), it can be concluded that the carbon amount in catalyst play an important role during the photocatalysis. Therefore, Pr_6O_{11}@C with various content of carbon was synthesized using different concentration of AR14 as carbon source and their photocatalytic properties are presented in Fig. S2. Pr_6O_{11}@C-1 is denoted as the Pr_6O_{11}@C sample prepared using 0.05 mM AR14 as carbon source, and Pr_6O_{11}@C-2 and Pr_6O_{11}@C-3 were obtained using 0.075 and 0.1 mM AR14 as carbon source, respectively. From Fig. S2, it can be found that appropriate amount of carbon in the sample is benefit for the photocatalytic efficiency. The reason may be that the adsorption capability of pollutant, the intensity of visible light absorption and the number of oxygen vacancies decrease when used less AR14 as carbon source, while the carbon will agglomerate on the surface of photocatalyst when used excess of AR14 as carbon source (Wang er al. 2018). Therefore, using 0.075 mM AR14 as carbon source is the best concentration to synthesize Pr_6O_{11}@C

with efficient photocatalytic efficiency.

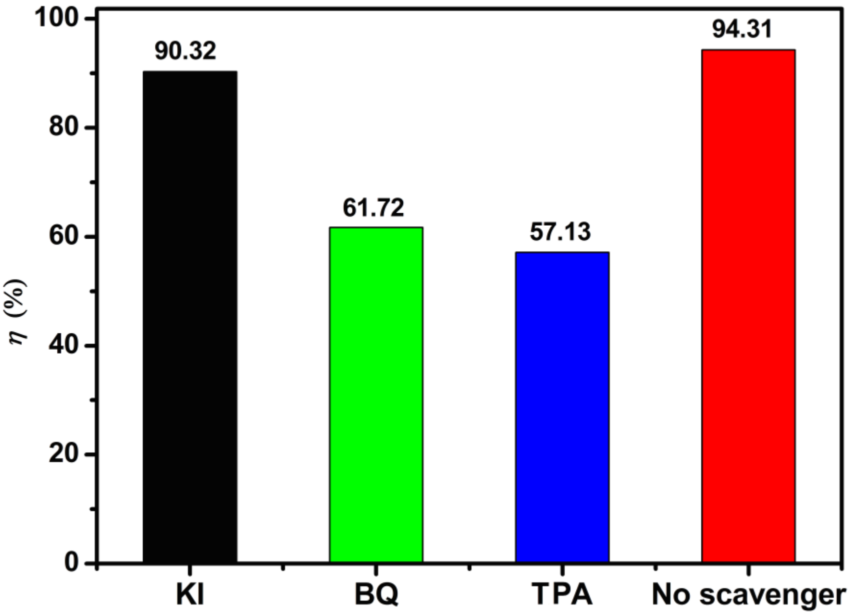

Fig. S3 Effect of KI, BQ, and PTA on the degradation of 0.3 mM AR14 over Pr_6O_{11}@C.

The determination of active species responsible for the photodegradation of AR14 is very important to understand the photocatalytic mechanism. Comparison experiments using KI as a quencher of positive holes (h^+), BQ as a quencher of superoxide radical ($O_2^{\bullet-}$), TPA as a quencher of hydroxyl radical (OH•) were carried out and the results are presented in Fig. S3. It can be found from Fig. S3 that KI almost did not affect the degradation rate of AR14, while the degradation efficiency of AR14 was decreased greatly when BQ and TPA were added in the reaction system, implying that $O_2^{\bullet-}$ and OH• are the main oxidative species responsible for the photodegradation of AR14.

3. References

Wang, H., Shang, J., Xiao, Z.L., Aprea, P., Hao, S.Y., 2020. Novel construction of carbon bonds in CeO_2@C with efficiently photocatalytic activity. Dyes Pigments 182, 108669.

Wang, X.T., Zhou, J.Q., Zhao, S., Chen, X., Yu, Y., 2018. Synergistic effect of adsorption and visible-light photocatalysis for organic pollutant removal over $BiVO_4$/carbon sphere nanocomposites. Appl. Surf. Sci., 453, 394-404.
